# Social cognition in a case of amnesia with neurodevelopmental mechanisms

**DOI:** 10.3389/fpsyg.2013.00342

**Published:** 2013-06-24

**Authors:** Angelica Staniloiu, Sabine Borsutzky, Friedrich G. Woermann, Hans J. Markowitsch

**Affiliations:** ^1^Physiological Psychology, University of BielefeldBielefeld, Germany; ^2^MRI Unit, Bethel Epilepsy CenterBielefeld, Germany; ^3^Institute for Advanced ScienceDelmenhorst, Germany; ^4^Center of Excellence Cognitive Interaction Technology, University of BielefeldBielefeld, Germany

**Keywords:** episodic–autobiographical memory, social information processing, theory of mind, hippocampus, hypoxia

## Abstract

Episodic–autobiographical memory (EAM) is considered to emerge gradually in concert with the development of other cognitive abilities (such as executive functions, personal semantic knowledge, emotional knowledge, theory of mind (ToM) functions, language, and working memory). On the brain level its emergence is accompanied by structural and functional reorganization of different components of the so-called EAM network. This network includes the hippocampal formation, which is viewed as being vital for the acquisition of memories of personal events for long-term storage. Developmental studies have emphasized socio-cultural-linguistic mechanisms that may be unique to the development of EAM. Furthermore it was hypothesized that one of the main functions of EAM is the social one. In the research field, the link between EAM and social cognition remains however debated. Herein we aim to bring new insights into the relation between EAM and social information processing (including social cognition) by describing a young adult patient with amnesia with neurodevelopmental mechanisms due to perinatal complications accompanied by hypoxia. The patient was investigated medically, psychiatrically, and with neuropsychological and neuroimaging methods. Structural high resolution magnetic resonance imaging revealed significant bilateral hippocampal atrophy as well as indices for degeneration in the amygdalae, basal ganglia, and thalamus, when a less conservative threshold was applied. In addition to extensive memory investigations and testing other (non-social) cognitive functions, we employed a broad range of tests that assessed social information processing (social perception, social cognition, social regulation). Our results point to both preserved (empathy, core ToM functions, visual affect selection, and discrimination, affective prosody discrimination) and impaired domains of social information processing (incongruent affective prosody processing, complex social judgments). They support proposals for a role of the hippocampal formation in processing more complex social information that likely requires multimodal relational handling.

## Introduction

Memory is divided according to time and content axes, respectively (Markowitsch and Staniloiu, 2012). Along the content axis, five long-term memory systems were described [procedural, priming, perceptual, semantic, and episodic–autobiographical memory (EAM) systems] (Tulving, 2005). These systems are considered to build up on each other ontogenetically and phylogenetically. EAM is considered the last ontogenetic and phylogenetic achievement (Nelson, 2003, 2005; Nelson and Fivush, 2004; Tulving, 2005). It is currently defined as being the conjunction of subjective time, autonoetic consciousness, and the experiencing self (Tulving, 2005). Autonoetic consciousness has been conceptualized in slightly different ways. Wheeler et al. (1997, p. 335) defined it as the “capacity that allows adult humans to mentally represent and to become aware of their protracted existence across subjective time.” Lemogne et al. (2006, p. 260) stated that autonoetic consciousness entails a “sense of self in time and the ability to relive subjective experiences from the encoding context by mentally traveling back in time.” Markowitsch proposed that autonoetic consciousnes is characterized by a superior ability to reflect upon oneself and distinguish oneself from the social and biological environment (Markowitsch, 2003; Markowitsch and Staniloiu, 2011a). While the latter definition of autonoetic consciousness might suggest a link between EAM and the dialectic of self and others (Suddendorf et al., 2009), the relationship between EAM (Tulving, 2005; Markowitsch and Staniloiu, 2012) and social cognition [theory of mind (ToM), empathy, simulation, social judgment, moral judgment] (Adolphs, 2010a) remains debated, and insufficiently explored experimentally.

Several authors proposed that EAM or autonoetic consciousness modulate an individual's capacity to make inferences about others' mental states and feelings, and distinguish these states from his or her own ones (Batson et al., 1996; Bluck et al., 2005; Saxe et al., 2006; Staniloiu et al., 2010a). Tulving (2005) remarked that Darwin's description of “moral being” had alluded to several features, which may be tied to morality, such as the capability for recollecting the past, the capacity for autonoetic consciousness and the ability to subjectively mentally travel in time, both into past and future. In this vein, difficulties with recollecting emotional events and/or autonoetic consciousness were propounded to exist in offenders with psychopathy, who feature impairments in empathy and affective ToM (Shamay-Tsoory and Aharon-Peretz, 2007; Craig et al., 2009). Croft et al.'s (2010) results suggested that severe EAM impairments due to neurological incidents may affect the updating of moral character judgments and subsequently may influence the way these individuals perceive and behave toward others. The authors compared in their study the performance of patients with bilateral damage to the ventromedial prefrontal cortex to that of patients with bilateral damage to the hippocampal formation (due to hypoxia/anoxia or herpes viral encephalitis) and that of a control brain-damaged group during a task that required the participants to make moral judgments about unfamiliar persons in two conditions (before and after being exposed to various social context scenarios). In contrast to patients with bilateral damage to the ventromedial prefrontal cortex, patients with bilateral hippocampal damage presented with severe impairments in conscious mnemonic processing, interfering with their everyday life. During the moral updating task, they furthermore demonstrated the largest amount of change in moral judgments after social scenario manipulations, compared to patients with bilateral damage to the ventromedial prefrontal cortex, who showed the least amount of change.

Social cognition (ToM) deficits were reported to co-occur with memory impairments in several psychiatric conditions, including dissociative (functional) amnesia (Corcoran and Frith, 2003; Reinhold and Markowitsch, 2007, 2008, 2009; Fujiwara et al., 2008). Kritchevsky et al. (2004, p. 224) described a patient with functional amnesia who after the onset of amnesia became “less aware of the feelings of other individuals.” He furthermore did not comprehend jokes anymore and “interpreted them literally” (Kritchevsky et al., 2004, p. 224). Interpersonal difficulties with family members have been reported to occur after the onset of dissociative amnesia and were partly attributed to an impaired ability to properly read the familiar/close others' mental states (Rabin and Rosenbaum, 2012; Staniloiu and Markowitsch, 2012; Markowitsch and Staniloiu, 2013).

Reinhold and Markowitsch (2007) formally assessed emotional processing and social cognition in two female adolescents (age 16 and 18, respectively) suffering from dissociative amnesia. They found that both patients were impaired on the German-language adaptation of the Reading the Mind in the Eyes Test (RMET) (Baron-Cohen et al., 2001; Fleck et al., 2002; Dziobek et al., 2006; Fujiwara et al., 2008) and emotional evaluation of ToM stories (Kalbe et al., 2007). In a case series of five patients with dissociative (functional) amnesia, three out of the four patients in whom the RMET was administered, showed performance deficits on it. Dissociative amnesia is however often accompanied by other psychiatric or medical comorbidities, such as major depressive disorder (Staniloiu and Markowitsch, 2012). One could furthermore argue that, even when the co-occurring symptoms of depression do not reach the threshold for a diagnosis of an affective disorder according to the international nosologies' diagnostic criteria (so-called subclinical symptoms of depression), they can still impact on ToM functions (Cusi et al., 2011). It was speculated that in some psychopathological conditions (Corcoran and Frith, 2003), which are known to have a neurodevelopmental basis, the co-occurrence of EAM and ToM deficits may reflect a developmental arrest of closely in time emerging neurocognitive functions, which are at that stage functionally interdependent (Perner, 2000; Bird et al., 2004; Nelson and Fivush, 2004). Other authors proposed that in certain forms of psychopathologies, an idiosyncratic way of making inferences about the mental states of others might take place, due to a failure of inhibition of own perspective and/or a poverty of models of the inner world of others (Newen and Schlicht, 2009). Functional neuroimaging studies suggested common (but also distinct) neural substrates for EAM and ToM (Spreng et al., 2008; Rabin et al., 2010; Spreng and Grady, 2010; Abu-Akel and Shamay-Tsoory, 2011). A recent investigation of healthy female participants revealed that the degree of neural overlap depends on the target person involved in the ToM task; when participants engaged in making inferences about the mental states of familiar others as opposed to unfamiliar others, they seemed to recruit more EAM-related brain areas, suggesting the use of a different cognitive strategy (despite identical task instructions) (Rabin and Rosenbaum, 2012).

By contrast, there are reports of patients with amnesia secondary to neurological brain insults incurred in adulthood with no detectable deficit on ToM functions (as assessed by using standardized laboratory tasks). Rosenbaum et al. (2007) investigated with a battery of widely employed tests (Stone et al., 1998; Castelli et al., 2000; Baron-Cohen et al., 2001; Dennis et al., 2001; Stuss et al., 2001) two patients with amnesia with onset after severe traumatic brain injury [patient K.C. (Rosenbaum et al., 2005) and patient M. L. (Levine et al., 1998, 2009)] and found that their performance did not significantly differ from that of 14 control participants on all measures. In addition, despite speculations that social cognition and EAM might depend on each other during early development (Perner, 2000; Nelson and Fivush, 2004), a recent study of an adult female patient (HC) with developmental amnesia showed that the patient performed within normal limits on a variety of standardized tests that assessed her capacity for ToM (Rabin et al., 2012). The applied testing battery consisted of: the False belief and the Faux pas tests (Stone et al., 1998), the 36 black and white photographs variant of the RMET (Baron-Cohen et al., 2001), the Sarcasm and Empathy Test (Dennis et al., 2001), the Visual Perspective-Taking and Deception Test (Stuss et al., 2001), and the Animation Test (Castelli et al., 2000).

The term developmental amnesia is a non-DSM-IV-TR (2000) terminology that designates a syndrome that occurs in childhood and is caused by relatively selective damage to hippocampi (usually resulting in more than 30–40% bilateral volume reduction of hippocampi in comparison to controls). Some involvement of the basal ganglia (bilaterally), thalamus (bilaterally), and right retrosplenial cortex, which was demonstrated in voxel-based morphometry studies had been reported as well (Vargha-Khadem et al., 1997, 2003; Isaacs et al., 2003). Consistent with the hypoxia-anoxia pathogenetic model (see below), in a patient with developmental amnesia white matter changes (e.g., thinning of the corpus callosum) were additionally remarked and in another cerebellar atrophy was noted (Vargha-Khadem et al., 1997; Gadian et al., 2000; Connolly et al., 2007).

The most common cause of relatively selective hippocampi damage is single or recurrent episodes of ischemic-hypoxia, which were reported to occur perinatally or in childhood until prepubertal period. Affected children can still acquire knowledge about facts and language skills depending on their intellectual ability that can range from low to normal, but show severe impairments in the episodic–autobiographical domain and everyday memory (Markowitsch and Staniloiu, 2012; Willoughby et al., 2012).

Most recently, studies of young adults with childhood developmental amnesia have focused on investigating the ability to imagine the future and on distinguishing between recollection/recall and familiarity/recognition (Kwan et al., 2010; Maguire et al., 2010). Little has, however, been devoted to a thorough investigation of social information processing in these cases, according to our knowledge.

Herein we provide a review, interpretation and critical discussion of results obtained with various tasks tapping on social cognition as well as other aspects of social information processing in a young adult male patient with amnesia with neurodevelopmental mechanisms. When we speak of social information processing, we use as guiding framework the classification described in Table 1 of Adolphs (2010a). In this table, Adolphs (2010a) depicted the following three stages of social information processing: *social perception* (perception of pheromones, face and speech perception, and perception of social touch and biological motion), *social cognition* (affective and cognitive ToM, simulation, empathy, social judgment, moral judgment), and *social regulation* (cognitive control, emotion regulation, monitoring/error correction, self-reflection, deception).

**Table 1:**
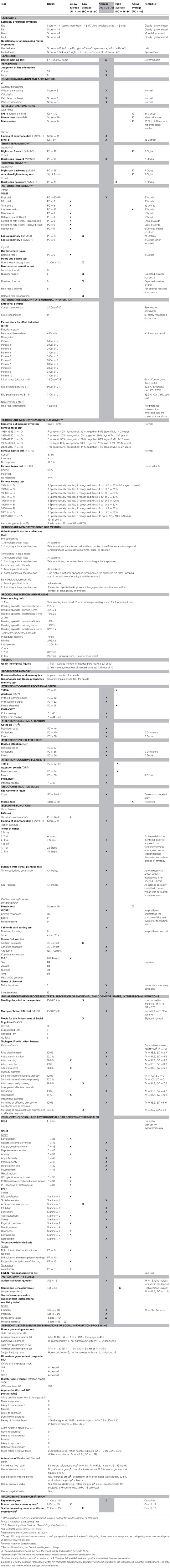
**Summary of test results**.

^1^TAP, Testbatterie zur Aufmerksamkeitsprüfung (Test Battery for the Assessment of Attention).

^2^WCST Wisconsin Card Sorting Test.

^3^TkS, Test für kognitives Schätzen (Test of Cognitive Estimation).

^4^Ss, subjects (Breitenstein et al., 1996)

^5^Replication study: Curci-Marino et al. (2004).

^6^Though ML partly showed results in tests of malingering which were indicative of malingering, these cannot be interpreted as malingering as he was usually poor in memory recall in general.

^7^Bremer Auditiver Gedächtnistest.

^8^Test zur Überprüfung der Gedächtnisfähigkeit im Alltag.

A T-score is a standard score that sets the mean to fifty and standard deviation to 10.

A percentile (PC) is the value of a variable below which a certain percent of observation fall.

Stanines are standard scores with a maximum of 9. Stanines 1–2 and 8–9 indicate significant deviations from normative data.

Stanines 1–3 on the subscale “Openness” of the FPI-R indicate elevated social desirability limiting the validity of the responses in the entire questionnaire. The gray column in the middle reflects average or normal scores.

## Case report

ML is a 29-year-old man who was 27-year-old at the time of the neuropsychological testing in our clinic. He is the oldest of three children, coming from a middle class family. Both of his siblings achieved higher education. His parents divorced when ML was a teenager. ML was born prematurely, at 33 weeks of gestation. After birth, he required a 2-week hospitalization for lung immaturity in a neonatal intensive care unit, where he received oxygen therapy. In terms of developmental milestones, ML began talking at age 13 months. Some stuttering was noted in childhood, but it ceased later on. From age 3 months until age 1 year, ML received physical therapy for problems with motor tone and muscle coordination. Sitting was delayed (he was older than 1 year when he was able to sit). He began walking at age 18 months and completed toilet training at age 3 years. At age 2 years, ML underwent another course of physical therapy. Both therapies followed the model developed by Vaclav Vojta (Sadowska, 2001) and were successful; however they were perceived by ML's mother as having been psychologically traumatizing. Clumsiness and other (usually transient) problems with motor skills were reported in other patients with developmental amnesia, who sustained hypoxic-ischemic events during the first year of life (Gadian et al., 2000; Vargha-Khadem et al., 2003). However, the reported difficulties were milder than the ones experienced by ML. Although postulated, a connection between the basal ganglia (and thalamic) damage and motor impairment in cases of developmental amnesia remained unclear (Gadian et al., 2000; Vargha-Khadem et al., 2003; de Haan et al., 2006).

ML entered kindergarten at age 4 years and was described during those time as being reserved and a daydreamer. Since age five ML has seen several health care providers and has been suspected of having several diagnoses such as Minimal Cerebral Dysfunction (MCD), autistic spectrum disorder, Asperger's syndrome, attention hyperactivity deficit disorder. None of these diagnoses was confirmed. ML's case may therefore reflect other cases of developmental amnesia from the literature, where an accurate diagnosis was far from being “straightforward” from the beginning (Gadian et al., 2000). As Gadian et al. (2000) remarked, it is not uncommon for problems with episodic memory–which typically become evident around age 5 or 6 years (in conformity with data on the ontogenesis of episodic memory; Nelson and Fivush, 2004) – to be initially attributed to attention deficits. Several motor tics and substantial problems with school performance were noted when ML was around the age of 7 years, shortly after he had entered the school. Problems with organization, memory for life events as well as performing several real-world memory tasks (Willoughby et al., 2012) were remarked. In the absence of a comprehensive and rigorous neuropsychological investigation at the time, ML's poor school functioning initially was however conjectured to reflect primary attention and concentration difficulties (Lebrun-Givois et al., 2008). On an interpersonal level, ML reportedly experienced difficulties establishing social contact with other peers during his early school years. He often imitated emotions or behaviors of others, instead of expressing his own feelings. Although not commonly reported, social and emotional difficulties were described in other patients with diagnoses of developmental amnesia by other authors (Picard et al., 2012).

ML later on outgrew his tics. Because of his persisting difficulties in school, ML was supervised by a school psychologist. In the following years, ML continued to experience memory impairments and lack of organization. Despite of otherwise good intellectual functions, he continued to fail school, which prompted his family to seek psychiatric help for him. Therefore, at age 16 years ML was brought by his mother for a comprehensive medical and neuropsychological assessment. Contrary to expectations, the neuropsychological assessment revealed above average attention and concentration abilities; verbal short-term memory was average, digit span was above average, his visual short-term memory was average and word fluency was within normal limits. ML was fully oriented. Verbal and visual memory performance after delays of half an hour were however impaired. ML displayed no evidence of distractibility and showed no heightened level of interference. There was no evidence of perseveration or apraxia, stereotypes, or tics. The neuropsychologist's recommendation was that ML should undergo memory rehabilitation training, although no clear diagnosis was provided.

A routine electroencephalography (EEG), which was performed at the time, yielded no evidence of seizures or epilepsy. Both computer tomography (CT) and magnetic resonance imaging (MRI) scans of the head were performed. The CT showed a discrete enlargement of the lateral ventricles downright. MRI revealed a small gliotic mass in the left thalamus and discrete prominence of the lateral ventricles and of the external cerebrospinal fluid space (increased sulcal cerebrospinal fluid). Hippocampal changes were not described and no comment was made about the significance of the enlargement of the lateral ventricles.

Over the years ML attended several therapies and he graduated from a special needs school. ML completed 10 years of schooling, repeating one school year. His school trajectory might therefore seem different than that of other patients with developmental amnesia described in the literature, who were reported to have attended mainstream schools (“albeit with considerable difficulties in some cases”) (Gadian et al., 2000, p. 505) (but, see also Bindschaedler et al., 2011). Incidentally, Picard et al. (2012) recently published two case reports of patients whom they diagnosed with developmental amnesia. One of those patients showed an atypical schooling pathway; she failed normal schooling and similarly to ML, she subsequently attended specialized school.

Since age 20 ML has received external help from a caretaker regarding planning, remembering important appointment dates, and managing finances. He has lived in a supervised setting since finishing school. ML's living situation again points to a difference between ML and other cases of developmental amnesia (Rabin et al., 2012). However it is worth mentioning that other patients with diagnoses of developmental amnesia were described to live in “protective environments” (though not in residential settings) (Picard et al., 2012). In contrast to his everyday memory impairments, ML has shown impressive special knowledge in some fields, which he has no problem to acquire and retrieve. He for example, has a very broad and detailed knowledge of special luxury goods such as watches and car brands. Furthermore, he can learn and remember very well pieces of music. ML joined a dance course 4 years prior to his assessment in our clinic. His substitute decision maker states that ML is a very good dancer and has an incredible ability to learn new forms of dancing. Similarly to other patients with amnesia after adult or early-onset hippocampal damage (Milner et al., 1968; Rabin et al., 2012), ML is aware of his memory impairment; he has learned to partially compensate for it by using strategies such as repetition and reliance on calendars to keep track of scheduled appointments.

On an interpersonal level ML's ability to interact with his peers has significantly improved since his school years. His substitute decision maker however voiced concerns about ML's heightened capacity to trust other people. In spite of being repeatedly told not to give money to other people (and carrying in his portemonnaie a visible note stating that), ML has continued to lend money to people without usually getting it back.

## Imaging findings

**Figure 1:**
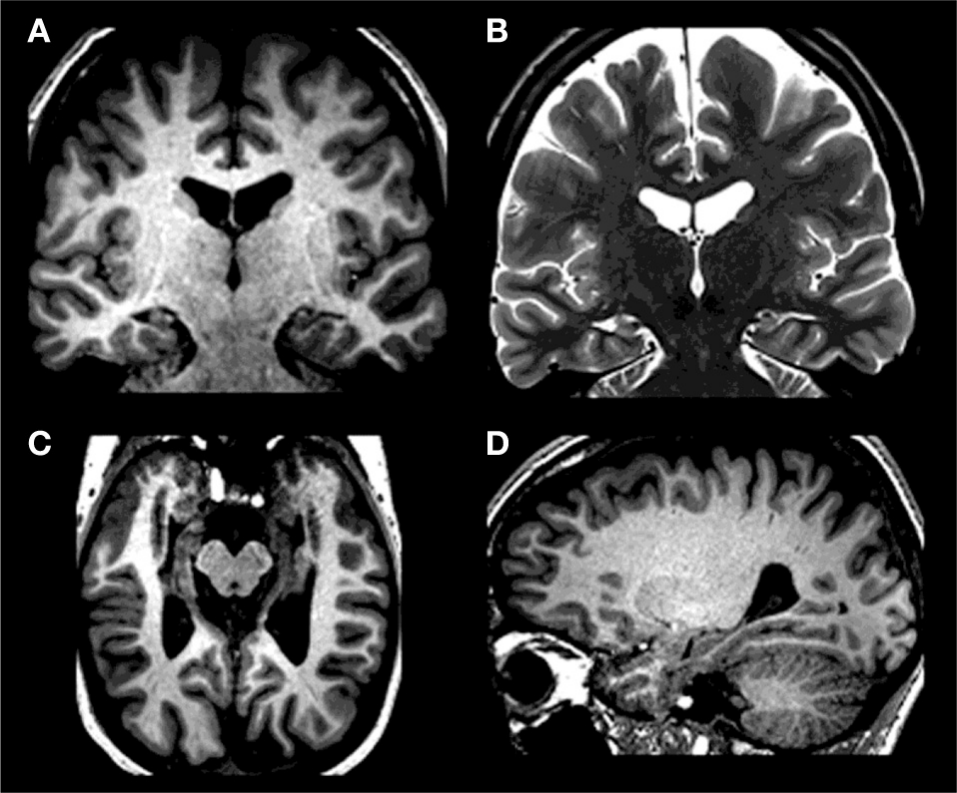
**Bilateral hippocampal atrophy in ML with T1-weighted images [(A) coronal; (C) axial; (D) sagittal] demonstrating reduced hippocampal size in all directions – in the absence of marked extrahippocampal atrophy; T2 weighted coronal image (B) demonstrating bilateral loss of internal structure – here: a further marker of bilateral hippocampal atrophy**. On clinical MRIs left side of the image is right side of the patient.

**Figure 2:**
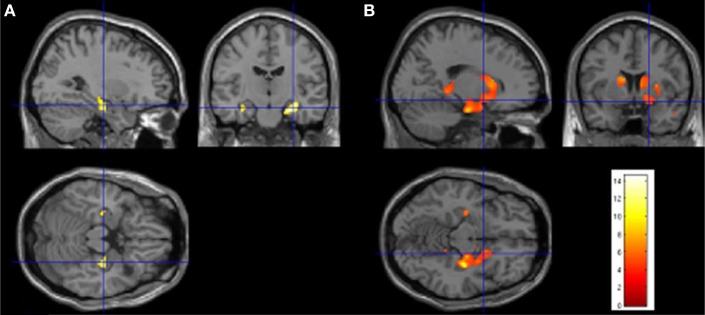
**Quantitative comparison of 3D T1-weighted images of patient ML with 10 age-matched control subjects using voxel-based morphometry (VBM; SPM8, Wellcome Institute, London, UK)**. For details regarding the method used here, please refer to Labudda et al. (2012). **(A)** Hypothesis-driven comparison within a hippocampal volume of interest demonstrates a marked reduction of gray matter volume within both hippocampi of the patient (*p* < 0.05, FWE) which has an anterior and right-sided preponderance (please note that *in SPM* the right side of the picture is the right side of the patient – *see crosshair*). **(B)** Using a whole brain analysis with a less conservative statistical threshold (*p* < 0.001, uncorrected), there are further reductions of gray matter, affecting amygdalae (bilaterally), bilateral dorsal striatum (mainly caudate and putamen, and to a certain extent the globus pallidus), portions of the ventral striatum (bilaterally), and posterior portions of the pulvinaris complex (also bilaterally), again with a right-sided preponderance.

ML underwent several structural MRI at different locations. The most recent imaging was performed when ML was age 29 years, with a 3-Tesla MRI scanner (Siemens Magneto Verio whole-body MRI system equipped with a head volume coil). The procedure was undergone in a neuroradiological center specialized for assessing patients with epilepsy pre- and post-operatively. The imaging data were evaluated by a neurologist with expertise in neuro-radiology (Friedrich G. Woermann). Visual inspection revealed grossly reduced (gray matter) density within hippocampi bilaterally (Figures 1 and 2). There was no evidence of pathology in the underlying parahippocampal region or other brain regions based on visual inspection. Using voxel-based morphometry (VBM; SPM8, Wellcome Institute, London, UK), a quantitative comparison of 3D T1-weighted images of patient ML with 10 age-matched healthy control participants was performed (for details regarding the method employed here, please refer to Labudda et al., 2012). The hypothesis-driven comparison within a hippocampal volume of interest demonstrated a marked reduction of gray matter volume within both hippocampi of the patient ML [*p* < 0.05, Familywise Error (FWE)], with an anterior and right-sided preponderance. Only when using a whole brain analysis with a less conservative statistical threshold (*p* < 0.001, uncorrected), we evidenced indices of further reductions of gray matter, affecting both amygdalae and basal ganglia (striatum, pallidum) – with a right-sided preponderance as well and pulvinar (bilaterally, but with a right-sided trend).

## Tests

### Neuropsychological assessment

The following tests were administered:

- Standardized tests for handedness and brain lateralization
  The Lateral Preference Inventory (LPI) for measurement of handedness, footedness, earedness, and eyedness (Ehrenstein and Arnold-Schulz-Gahmen, 1997), and the Questionnaire for measuring motor asymmetry (Reiss and Reiss, 2000). This last test contains 12 queries for assessing handedness and footedness.
- Standardized tests for the estimation of intelligence and overall cognitive status
  Abbreviated Wechsler Adult Intelligence Test-Revised (Block test and Picture Completion test) (Dahl, 1972). MWT-B or Mehrfachwahl-Wortschatz-Intelligenztest-B (Lehrl, 2005), a German version of the National Adult Reading Test NART (Nelson, 1982). The reasoning and rule recognition subtest from Leistungsprüfungssystem (LPS-4; Horn, 1983). The Mosaic Test from the revised Hamburg Wechsler Intelligence Test (HAWIE-R; Tewes, 1991), a German-language adaptation of the Wechsler Intelligence Test for Adults-Revised (WAIS-R) (Wechsler, 1981). Matrices Test from Wechsler Intelligence test for Adults (Aster et al., 2006). Commonalities finding test from HAWIE-R (Tewes, 1991).
- Standardized tests for the evaluation of attention, concentration, and processing speed
  Trail Making Test A and B (TMT-A + TMT-B; Lezak, 1995; Reitan, 1958); Attention Index of the German version of the Wechsler Memory Scale-Revised (WMS-R; Härting et al., 2000); the subtests Alertness, Selective Attention, and Divided Attention of the Test Battery for the Assessment of Attention or Testbatterie zur Aufmerksamkeitsprüfung (TAP) (Fimm and Zimmermann, 2001). The TAP is a computer-based test that assesses attentional performance based on reaction times. The Alertness subtest provides a measure of general slowing. The Selective Attention subtest is a go/no-go task, during which the participant has to selectively react to a group of stimuli, but not to others, and to inhibit a dominant response. The Divided Attention subtest is a “dual task” paradigm that assesses the ability to flexibly switch attention between two ongoing tasks (Fujiwara et al., 2008).
- Standardized tests for evaluation of short-term memory and working memory
  Wechsler Memory Scale-Revised; digit span and block span forward and backward; adaptive digit ordering test (Hoppe et al., 2000). In this last test digits which are presented in random order have to be recalled in ascending order (e.g., 4–3–9–3 should be recalled as 3–3–4–9).
- Standardized tests for the evaluation of constructional functions and planning
  Copy administration of the Rey-Osterrieth Figure Test (Osterrieth, 1944; Lezak, 1995); Benton Visual Retention Test (Lezak, 1995; Spreen and Strauss, 1998); Burgau Little Verbal Planning Test (*Burgauer Kleiner Verbaler Planungstest*; von Cramon and Zihl, 1989); Test of Cognitive Estimation (TkS; Brand et al., 2002, 2003a,b). The Benton Visual Retention Test taps on many different abilities such as visuo-spatial perception, visual and verbal conceptualizations, and working memory at the border to long-term memory. The Burgau Test requires the planning of a time-based sequence in which several transactions (going shopping) have to be organized within a given time. The TkS is a German-language test for cognitive estimation (Shallice and Evans, 1978), during which participants are requested to estimate numbers, weights, heights/lengths, and time durations.
- Standardized tests for the evaluation of the verbal and non-verbal explicit anterograde long-term memory
  Wechsler Memory Scale-Revised (Härting et al., 2000); Verbal Learning Memory Test (VLMT) (Helmstaedter et al., 2001); Rey-Osterrieth Figure; Copy trial followed by delayed recall after 30 min (Lezak, 1995); the Doors Test (visual recognition) of the Doors and People Test (Baddeley et al., 1994; Adlam et al., 2009); Rivermead Behavioral Memory Test (Wilson et al., 1985). The VLMT requires the recall of a list A of 15 presented words for 5 trials, the recall of a second (interference) list B, then again recall of the list A, and recall of it after 30 min delay, and finally the recognition of words of list A from words belonging to lists A and B and to none of the two. The Doors test requires the visual recognition of 12 easily and 12 difficultly to discriminate doors, each from an array of 4 doors. And the RBMT contains a number of subtests assessing retrospective and prospective visual and verbal memory functions.
- Tests for the evaluation of retrograde memory (standardized or qualitative)
  Semantic Knowledge Test [Semantic Old Memory Inventory; Schmidtke and Vollmer-Schmolck, 1999 (qualitative description)]; Bielefeld Autobiographical Memory Interview (BAGI; standardized; partly based on the Autobiographical Memory Interview of Kopelman et al., 1990; Fujiwara, 2004; Fujiwara et al., 2008; Fast et al., 2013; Famous Faces Test; standardized; see Jänicke, 2001), Famous Terms, Famous Events and Famous Names Tests [(qualitative descriptions); Leplow and Dierks, 1997; Markowitsch, 2003; Fujiwara et al., 2008]. In the Semantic Knowledge Test general facts are asked (“What is the currency in Switzerland?”), in the BAGI two episodes with time, place, emotional involvement are requested from all periods of the past life, divided into 5- or 10-year epochs.
- Test for evaluation of prospective memory – qualitative description (Knight et al., 2010; Staniloiu and Markowitsch, 2012)
  Recalling to perform an intended future action, in particular to ask at the end of the testing for a personal object that the examiner had borrowed from the patient and hid in the examining room, in response to a pre-specified cue, namely the end of the testing. “The Grasshoppers and Geese Prospective Memory Test” (Lanting et al., 2010, 2011), which was developed for ethnically diverse individuals, comprises a task instruction that is embedded semantically. The test requests that the patient reminds the examiner to perform a task when an external (verbal) cue is delivered. If the patient does not respond to the cue, a series of three verbal prompts are provided. Scores range from 0 (no prompts required) to 4 (no recall to perform the action after all three prompts).
- Tests for the evaluation of priming (standardized) and procedural memory (qualitative description)
  Mirror Reading Test (von Cramon et al., 1993; Borsutzky et al., 2008, 2010), Gollin Incomplete Pictures Test (Gollin, 1960; Markowitsch et al., 1993; von Cramon et al., 1993).

For the Mirror Reading Test the version that is described in Borsutzky et al. (2008, 2010) was used. This variant allows a differentiation between priming and procedural memory. It comprises a series of 15 cards each consisting of two German words in mirror writing (30 words in total, all words with 8 to 10 letters) which are visually presented to the participants. Participants are asked to read the words as quickly and accurately as possible. Upon correct reading of the two words of one card, the next card is presented. The time by which subjects read both words correctly, constitutes the reading time measure. If they read a word incorrectly, they are told so. However, the correct word is not provided. In addition, all incorrect responses are counted. After a delay of 30 min a surprise second trial is administered. Herein, 10 words (on five cards) are identical with the first trial, 10 words are new and 10 words are also new, but similar in orthography to 10 words of trial 1 (e.g., “Explosion” and “Exkursion”). The sequence of cards is randomized for both trials. Improvement in reading speed from trial 1 to trial 2 of the recurring words serves to measure priming performance (“Priming”). Improvement in reading time of new words is used for an index of skill acquisition, i.e., procedural learning (“Procedural Memory”). Those words of trial 2 that are orthographically similar to words of trial 1 (“Interference”) are created to induce interference in reading. We assume that due to these similarities in orthography with the words of trial 1, subjects might be disturbed in automatic reading processes. In trial 2 participants may initially tend to recognize the similarly looking words of trial 1 due to priming effects, but during reading, they would notice most discrepancies and would then have to execute a more effortful analysis. On the one hand, this may lead to longer reading times. On the other hand, if subjects experience deficits in suppressing the activated, but irrelevant memory trace (i.e., the “Interference” words of trial 1), they may produce more mistakes in reading of “Interference” words in trial 2 compared to “Priming” and “Procedural Memory” items.

In the Gollin Incomplete Pictures Test 20 pictures are presented, each picture containing a single familiar item (tree, ship, rabbit, etc.). Each item is given in 10 versions from a barely suggestive drawing (just a few dots or line drawings) to complete figure drawing. The participant has to tell what he or she thinks is to be seen in the drawing. Usually healthy participants recognize the item between versions 5 and 6, when given the first time, but much earlier (version 2 or 3), when it is given the second time.

- Tests for the assessment of executive functions, problem solving, and cognitive flexibility
  Trail Making Test-B (Lezak, 1995); Tower of Hanoi (Borys et al., 1982; Spitz et al., 1982; Lezak, 1995); California Card Sorting Test (Delis et al., 1987, 1992); problem solving test of Cronin-Golomb et al. (1987a,b); Wisconsin Card Sorting Test (Nelson, 1976); Game of Dice Task (Brand et al., 2005; Brand and Markowitsch, 2010). (All tests but the problem solving test of Cronin-Golomb are standardized.) In the problem solving test participants are given 17 sheets of paper, one after another; each sheet shows a drawing on the left side of the paper and three drawings on the right side (e.g., a crescent moon on the left side and a penguin, a woodpecker, and an owl on the right side). The participant should tell which of the three drawings on the right side best matches the drawing on the left side; we provide a qualitative description of performance on this test.

The Game of Dice Task is presented via computer. It is a fictitious gambling situation with explicit rules for virtual gains, losses, and winning strategies, which assesses decision-making under risk. A virtual single dice and a shaker are employed. Before throwing the virtual dice for 18 times, participants each time have to choose a single or a combination of 2, 3, or 4 numbers. Each choice is associated with different gains or losses. Participants get visual and acoustic feedback for their former decision.

- Standardized tests of verbal fluency
  Controlled Oral Word Association Test [COW fluency tasks (FAS); Golden et al., 2002]; Supermarket task (word production) (Calabrese and Kessler, 2000); Boston Naming Test (Kaplan et al., 1983; Lezak, 1995; Spreen and Strauss, 1998; Golden et al., 2002); the German version of the Stroop Test (Farbe-Wort-Interferenz Test/FWIT; or CWIT for Color Word Interference Test; Stroop, 1935; Bäumler, 1985).
- Standardized tests for calculation
  Zahlenverarbeitungs- und Rechentest (ZRT; Kalbe et al., 2002). In this test various simple and more complex calculations have to be done (additions, subtractions, multiplications, divisions).
- Standardized tests for evaluation of malingering tendencies
  Tests of memory malingering/effort [*Rey 15-Item-Test* (Lezak, 1995); tests from the *Test Battery for Forensic Neuropsychology* (*TBFN*; *Bremer Auditory Memory Test; Test for Assessing Memory Ability in Everyday Life*) (Heubrock and Petermann, 2000)]. In the TBFN subjects listen to a number of sounds (e.g., cackling of geese, honking) and later have to name them, or they listen to statements and later get for each statement a choice of four alternatives what was said (e.g., whether a meeting was at 10, 12, 2, or 4 o'clock).
- Tests for evaluation of emotional processing
  *Florida Affect Battery* (translated as *Tübingen Affect Battery*; Bowers et al., 1991; Breitenstein et al., 1996; standardized test); Emotional Pictures Test (von Cramon et al., 1993; performance on this test was qualitatively compared with the performance of the healthy control group and patient HI with septal nuclei damage described in von Cramon et al., 1993); Picture Story for Affect Induction (BAd; Temizyürek, 2003; qualitative description); Interpersonal Reactivity Index (Saarbrücker Persönlichkeitsfragebogen/Saarbrücken Personality Questionnaire; standardized; Beven et al., 2004; Paulus, 2012). In the Emotional Pictures Test 40 photographs with scenes of a neutral, positive, or negative content (e.g., shooting at a person, kissing another person) are presented and after half an hour the 40 original pictures had to be recognized out of 80 photographs. Control healthy participants (von Cramon et al., 1993) performed at a level of 95% correct for emotional and 88.3% correct for neutral pictures in the Emotional Pictures Test.

In the BAd two versions of a story about a family (mother, father, child) are shown – in the neutral version a day in their life is shown in pictures without that the child is involved in a car accident; in the emotional version the child is involved in a car accident. While watching the pictures the participant also listens to a male voice providing a narrative for each picture. The two variants of the story are identical for the first and last third of the pictures, but differ for the second third.

- Standardized tests for mood, personality and psychopathological, and psychological load screening
  Beck Depression Interview-II (BDI-II; Hautzinger et al., 2006); The Symptom Checklist Revised or SCL-90R (Hessel et al., 2001); Freiburg-Personality-Inventory-Revised (FPI-R; Fahrenberg et al., 2001); The German version of the Toronto-Alexithymia-Scale (Kupfer et al., 2001); Autism-Spectrum-Quotient (Baron-Cohen et al., 2006); Cambridge Behavior Scale (de Haen, 2006); Eigenschaftswörterliste EWL-N (List of personal adjectives) (Janke and Debus, 1978). The SCL-90R assesses psychiatric symptom load and psychological distress. It has nine subscales, such as somatization, obsessive-compulsiveness, interpersonal sensitivity, depression, anxiety, anger-hostility, phobic anxiety, paranoid thinking, and psychoticism. The general psychological distress level is estimated based on a global severity index, which is derived from all subscales. The FPI-R provides an assessment of personality along 12 dimensions: life satisfaction, social orientation, motivation to achieve, inhibition, excitability, aggressiveness, stress, physical complaints, health worries, openness, extraversion, and neuroticism. The EWL-N is a multidimensional self-administered scale, describing the actual well-being. It contains 15 subscales, assessing activity, concentration, passivity, fatigue, extraversion, introversion, self-confidence, mood elevation, excitability, interpersonal sensitivity, irritability, anxiety, depressive tendency, dreaminess, and numbness. The scale was given to both ML and his mother who were both requested to choose the personal adjectives that matched ML's characteristics best.
- Standardized test for visual perception
  Judgment of Line Orientation (Lezak, 1995; Mitrushina et al., 2005).
- Tests for social information processing

#### Tests for social perception (perception of face, speech perception)

“Tübinger Affekt Batterie,” a German-language version of the Florida Affect Battery (Bowers et al., 1991; Breitenstein et al., 1996) was administered.

#### Tests for social cognition (theory of mind, empathy)

“Augen-ToM-Test,” a 24 photographs containing German adaptation of the well-known RMET (Baron-Cohen et al., 2001; Fleck et al., 2002; Reinhold and Markowitsch, 2007; Fujiwara et al., 2008) was administered (for a detailed description of the version of this test, see Dziobek et al., 2006). During the task only the eye regions of female (12) and male (12) faces are presented. The participant has to choose one of the four verbal descriptors (words) that she or he thinks it describes the best the mental state of the character. For a comparison group, we referred to age and educational matched data from Reinhold and Markowitsch (2007) and Dziobek et al. (2006). Mean scores and standard deviations are presented in Table 1.

The Multiple-Choice-ToM-Test (MCTT; Kalbe et al., 2001; Adenauer et al., 2005) version that we used requires the participants to read 16 short stories. (The test exists in two versions, one with 30 short written stories and another one with 16 stories. We provide a qualitative description of the performance on the abbreviated version of the MCTT). After each story (see example below) the participant has to make inferences about the mental states of a character of the story by resorting to a forced multiple choice format with four possible answers (only one right answer). The multiple choice format enables differentiation of three different types of mistakes: (a) mental states inferences that are “excessive”; (b) mental state inferences that are “too positive”; (c) choice of the distractor answer that reflects a neutral answer (i.e., a non-mental state inference such as physical causation).

Example of a story from MCTT:

Die Bananenschale (The banana skin; translated from German):

Joseph slips on the footpath on a banana skin and falls down. A woman close to him starts to laugh loudly. What does Joseph think?

1. Such a silly woman. She should keep her mouth shut.
2. Well, she is in a good mood.
3. She definitely smashed the banana skin purposefully to this place.
4. The woman wears a nice skirt.

The movie for the assessment of Social Cognition (MASC) (Dziobek et al., 2006; Fleck, 2008) requires the participants to watch a video about four individuals in their mid-thirties who gather for a dinner party. The movie is 15 min long, but it is paused and the participants have to answer questions concerning the film characters' emotions, thoughts and intentions. Therefore the test session lasts around 45 min. The test has been applied to healthy participants, patients with Asperger syndrome (see Dziobek et al., 2006) and patients with schizophrenia (Fleck, 2008). For comparison groups, we referred to the study of Dziobek et al. (2006). Mean scores and standard deviations are presented in Table 1.

The animation of Heider and Simmel (1944); Lück (2006), and Curci-Marino et al. (2004). The animated and silent short movie created by Heider and Simmel shows three geometrical figures (a large triangle, a small triangle and a small circle), which are moving in the neighborhood of a rectangle. In our study, we used a procedure similar to that of Curci-Marino et al. (2004), who applied this test to 40 German-speaking participants (15 women and 25 men) recruited from a technical university with a mean age of 24.3 years, in an attempt to replicate the original results of Heider and Simmel (1944). The movie was shown twice on a computer screen. As Heider and Simmel, we used a general task, such as “describe in writing what you saw in the movie.” The text produced by the participant was then analyzed in a manner similar to that of Curci-Marino and colleagues and the results were qualitatively compared to those obtained by Curci-Marino and colleagues. We recorded the number of total words used and analyzed the use of animate nouns versus nouns designing geometrical figures, the description of internal states, the use of physical verbs versus animate verbs.

Understanding humor (Happé et al., 1999; Shammi and Stuss, 1999; Stuss and Levine, 2002; Uekermann et al., 2006; Bodden et al., 2010a) was qualitatively investigated. In the Humor task that we used, the participant is shown 20 cartoons from popular magazines. Similarly to the design of Happe et al. (1999), the cartoons are grouped in two conditions: 10 ToM cartoons and 10 non-mentalistic cartoons (the latter involved a physical anomaly). Cartoons are randomly shown, one at a time, on a computer screen. The participant is told to look at each cartoon and announce when he understood the joke. He then is asked to try to explain to the experimenter why each cartoon is funny. In addition to providing an explanation, the participant is asked to provide a subjective funniness rating for each cartoon. The mean times needed for processing the ToM cartoons and non-ToM cartoons are recorded (Bodden et al., 2010a).

#### Tests for social cognition (social judgment)

The *Approachability Task* (Adolphs et al., 1998) assesses the ability to make social judgments of other people. For this study we used an adaptation of the original task of Adolphs et al. (1998), which had been employed by Bellugi et al. (1999) to test 26 participants with Williams syndrome (WMS; mean age 23.6) and 26 healthy participants of similar age and gender ratio (mean age 25.5). Similarly to Bellugi et al. (1999) we used 42 black and white photographs of unfamiliar human faces, which were taken out of a pool of 100 original stimuli (Adolphs et al., 1998); 21 of these photographs depicted faces for which normal controls had given more negative ratings and 21 showed faces for which normal controls had given more positive ratings. The photographs were shown to ML one at a time, in random order. Without time constraints, ML had to rate the approachability of the face stimulus on a five point Likert type scale, which ranged from −2 (very unapproachable) to +2 (very approachable), by indicating how much he would approach and strike up a conversation on a street with the person whose face was depicted in the photograph. ML's performance was qualitatively compared to the results of Bellugi et al. (1999).

#### Tests for social cognition (moral judgment)

We used variations of the *Ultimatum* and *Dictator Games* which deviated from standard tests in several respects (Koenigs and Tranel, 2007). In the *Ultimatum Game*, ML acted as the responder. The starting capital was 100 Euros (Bolton and Ockenfels, 1998; Vieth, 2003; Oosterbeek et al., 2004). However, in contrast to the standard *Ultimatum Game*, participation was at the end of the experiment compensated with a fixed amount regardless of responses on the task (Koenigs and Tranel, 2007). Furthermore, in this variation, the examiner knew the proposers' identity and the offers were predetermined by the experimenter (Koenigs and Tranel, 2007). In the variation of the *Ultimatum Game* we used, the starting capital was 100 Euros; again, a fixed compensation at the end of the experiment regardless of responses on the task was offered. ML acted as dictator, deciding how much of the capital to keep. Again, the examiner knew both the identity of the “dictator” and “recipient.”

#### Tests for social regulation (cognitive control, emotion regulation, monitoring/error correction, deception)

Game of Dice Test (Brand et al., 2005; Brand and Markowitsch, 2010); Interpersonal Reactivity Index *(Saarbrücker Persönlichkeitsfragebogen/Saarbrücken Personality Questionnaire;* Beven et al., 2004; Paulus, 2012); tests for evaluation of malingering tendencies (see above). The Game of Dice Task was described above under tests for executive functions. The personal distress scale of the Saarbrücken Personality Questionnaire contains queries which tap on emotional regulation.

Prior to starting the testing process, Mr. N. was asked a series of general interview questions, to assess for problems with awareness and orientation.

## Testing results and interpretation

### General behavioral observations

ML came to the assessment accompanied by his surrogate decision maker. Informed written consent was obtained for the participation in the study and publication of the report. The study adhered to the declaration of Helsinki.

ML was eager to participate and to show what he is able to do. In the following we will describe results from testing him over a period of about 8 h (with breaks). A summary of ML's testing results is provided in Table 1.

### Laterality

ML in general was lateralized to the right, though he used his left hand for writing and other ways of motor performance *(Laterality Preference Inventory*). In the *Questionnaire for Measuring Motor Asymmetry* he provided evidence for a symmetrical foot use and more frequent use of his left hand.

### Language and word knowledge

ML's knowledge of terms and words was good *(Boston Naming Test*). He just failed to name *asparagus* and did not know the exact terms for *yoke* and *abacus.*

### Visual perception

Visual perception, as tested with the *Line Orientation Test*, was within normal limits.

### Attention, concentration, and processing speed

In all test of attention, concentration, and processing speed ML gained at least within normal limits situated scores. In the *Trail Making Test A and B* his performance was above average; testing results for reaction speed and phasic alertness were indicative of above average performance as well. Performance on divided attention and selective attention tasks was within normal limits (*Test Battery for the Assessment of Attention or TAP*). Stroop test performance was within normal limits as well.

### Number calculation and arithmetic

Number processing and arithmetic were perfectly normal.

### Intelligence and overall cognitive status

Several assessment procedures were used. In a test for estimating his verbal intelligence (MWT) ML was average. Also his performance in finding commonalities was average. On the other hand in non-verbal logical thinking ML was quite superior (IQ = 130). In other non-verbal tests of IQ his performance was superior to normal.

### Short-term and working memory

Performances on digit span forward and backward were above average in both numerical and visual tests. The surprising exception was an only average performance in visual block span forward (while the performance on visual block span backward was above average). Also ML was not prone to interference.

### Visual-constructive skills

In the Copy administration of the *Rey-Osterrieth Figure* ML reached points that situated his performance within normal limits. In the *Mosaic test* he was flawless.

### Long-term memory

A large number of tests were applied to assess ML's long-term memory abilities. They can be divided into tests of anterograde, retrograde, and prospective memory, episodic–autobiographical, semantic, procedural, and priming memory (cf. Markowitsch and Staniloiu, 2012).

#### Anterograde memory

On all tests of anterograde memory, ML was below the level of normals (the only exception was that in the interference list of the *VLMT* he behaved substantially above average; this result can, however, be interpreted as reflecting his inability to properly acquire the main list of words, which was given prior to the interference list). Especially when it came to delayed recall of information he scored repeatedly at percentile 0 (*VLMT*, Logical Memory Subtest of the *Wechsler Memory Scale-Revised*, and *Rey-Osterrieth Figure, delayed recall*). Also in the *Benton Visual Retention Test* his performance was below average.

In more easy tasks of visual recognition he encountered much less problems (simple doors of the *Doors and People Test*) (Adlam et al., 2009). Also in recognition memory tests with emotional material he performed sub-average, but not as poorly as in free recall tests involving emotional material. During the Emotional Pictures Test, ML recognized only 60% of the emotional pictures, which places his performance far below that of the control group described in the study of von Cramon et al. (1993). Even more surprising was the high number (9) of falsely recognized distractors, i.e., of pictures he had not previously seen (cf. Table 1).

With respect to ML's performance in the picture story for affect induction (BAd), there were no differences in the free recall task of emotional versus non-emotional variant of the story. This suggests that his mnemonic performance did not benefit from the potential emotional enhancement (Markowitsch and Staniloiu, 2011b). With respect to the recognition task of the emotional version of the story, ML recognized 18 out of 28 details (64%) from the first pictures (1–4), 9 out of 21 (42.8%) details from the pictures presented in the middle (5–7), and only 7 out of 21 (33.3%) details from the pictures presented at the end (8–10). The pictures 5–7 are accompanied in the emotional variant of the story by an emotional narrative delivered by a male voice, while the other pictures are accompanied by a neutral narrative, which is identical for both the “emotional” and “neutral” versions of the story. In the study of Temizyürek (2003) that included 30 participants (mean age: 33.8 + 8.9 years; IQ = 108), the percentage of recognized details from the “emotional” variant of the story was 83% for the first pictures (1–4), 77% for the middle pictures (5–7), and 72.7% for the pictures presented at the end (8–10). The lower recognition of details from the last compared to the first part of the story in the case of ML may reflect forgetting in the context of cognitive overload (Corkin, 1984; Markowitsch et al., 1993).

#### Retrograde semantic memory recognition

In tests of retrograde semantic memory recognition ML, on the other hand, was principally normal (*Semantic Old Memory Inventory, Famous Faces Test, Famous Names, Famous Terms, and Famous Events Tests*).

#### Retrograde autobiographical memory recall

As it was evident from the *Autobiographical Memory Inventory*, ML was unable to recall any personal events apart from one outstanding event, where his father made a suicide attempt by jumping out of the window. On the other hand, he could list autobiographical semantic facts (date of birth, place of birth, schooling, and the like).

#### Prospective memory

His prospective memory appeared impaired (*Rivermead Behavioral Memory Test; “The Grasshoppers and Geese Prospective Memory Test”*). In the *Rivermead Behavioral Memory Test* ML did not spontaneously recall to ask back for the loaned item. He required several very explicit cues. After each cue he responded “Yes, there was something,” without knowing what. When given alternatives, he finally selected the right response.

On the *Grasshoppers and Geese Prospective Memory Test* ML did not perform the required action after all three prompts. (When extending the prompts to six – which is against instructions – he finally recalled the action he needed to perform.)

#### Procedural memory and priming

His procedural and priming skills appeared to be intact.

### Malingering

Tests of malingering partly yielded results which – for an individual with normal memory capacity – would be indicative of malingering. As ML was quite deficient in memory recall of new material in general, these test outcomes cannot be interpreted as providing evidence for feigning or malingering (Sollman and Berry, 2011).

### Executive functions and problem solving abilities

ML's problem solving and executive abilities were to a large extent within normal limits. Cognitive estimation measures were impaired (TkS). In the TkS, ML showed similar deficits as patients with Korsakoff's syndrome (Brand et al., 2003a,b). He exhibited deficits in estimating dimensions “weight,” “quantity,” and “time,” whereby time and weight estimations were the most deteriorated. Size estimation was normal. Deficits in the TkS time items had been speculated to depend on timing deficits combined with remote memory impairment (Brand et al., 2003a). [In the time estimation task used here the participant was asked to estimate the duration of specific events (e.g., duration of a morning shower) without experiencing them in the test situation itself (Brand et al., 2003a).]

In the *Concept Comprehension Test* (Cronin-Golomb et al., 1987a,b) ML's performance was sub-average for abstract, but within normal limits for concrete concepts (Martins et al., 2006). Performance on verbal *FAS* was below average as well, resembling other reports on patients with developmental amnesia (Temple and Richardson, 2006).

### Social information processing: perception of emotional and cognitive states and interpersonal situations

In the German adaptation of the *RMET* ML was only slightly impaired. In terms of qualitative findings, he required a relatively long time to respond. He made eight mistakes, but had no difficulties with reading fear. He rated two female eye pairs as belonging to a male.

In the MASC his performance was again only slightly impaired. ML's performance in this task seemed much closer to that of the 20 healthy controls from the study of Dziobek et al. (2006) (34.8 + 2.7) than to that of the 19 patients with Asperger's syndrome (24.4 + 5.9) from the same study (cf. Table 1).

In the *Florida (Tübingen) Affect Battery* ML displayed below average performance in the more complex subtests (affect naming, affect matching, affect prosody naming, detecting incongruent affect prosody, and matching face expression to affective prosody. In the visual affect naming subtest ML incorrectly designated the emotion of “fear” as being “anger” on two occasions. In the affect matching subtest, he made two mistakes, pertaining to the emotions “fear” and “happy,” respectively. In the naming of affective prosody, he made two errors, pertaining to the emotion of happiness (which he interpreted as “neutral”) and “fear.” In the subtest requiring matching emotional faces to affective prosody he made one mistake, which involved the emotion of “fear.” The most impaired was his performance on incongruent affective prosody, in which the semantic context was incongruent with prosody (Breitenstein et al., 1998; Snitz et al., 2002; Paulmann et al., 2008; Ward et al., 2012). While the degree of impairment detected in ML's performance should be interpreted with caution given the well-known ceiling effects of the *Florida Battery Test*, the qualitative description of these impairments is intriguing, when it is corroborated with findings from other tests, such as the Approachability Task or EWL-N (see below).

### Other tests of social information processing

The results in the *Humor Appreciation Test* (cartoons) were ambivalent. In the variation of *Ultimatum Game* ML accepted all the offers, including the minimal value (1 Euro) and in the *Dictator Game* he offered 15 Euros, which represents 15% of the starting capital. Although this behavior is difficult to be accurately interpreted due to the non-standardized format of testing that we used, there are, in our opinion some hints of abnormal fairness attitudes (Scheele et al., 2012; Baumard et al., 2013). Incidentally abnormal fairness attitudes and socio-economical decisions have recently been related to amygdala dysfunction (Scheele et al., 2012).

Results of the *Approachability Task* are indicative of either heightened capacity for trust, or hypersociability (see Bellugi et al., 1999; Martens et al., 2009), or alternatively, decreased aversiveness (such as in patients with amygdala damage; Adolphs et al., 1998, 1999). In the task of Heider and Simmel, ML used a total of 83 words in his description, within the range of healthy participants (Curci-Marino et al., 2004). However, he did not use any animate noun and gave no description of internal states. This finding is interesting in the light of data from Curci-Marino et al. (2004). This study (which was conducted in a technical university and without any explicit indication that it was a psychological study) failed to replicate several findings of Heider and Simmel (1944). The latter had reported a high use of animate nouns in their sample (97.1%). In the sample of Curci-Marino et al. (2004), 27 (59.3% men) out of 40 participants (67.5%, *p* < 0.05) did not use any animate nouns; only 13 participants out of 40 did use animate nouns (32.5%). The description of internal states was done by nine participants (22.5%, *p* < 0.01). Out of these participants six were men. Physical verbs were used by 39 participants and 38 participants described the moves of the figures with animated verbs among others. In contrast to Curci-Marino et al. (2004), the administration of the animation task of Heider and Simmel was done in our study as part of the psychological testing, which in theory could have offered ML a performance advantage.

### Psychiatric ratings, emotions, and personality

The screening instrument *Beck Depression Inventory* did not yield scores suggestive of an affective disorder. ML did not show autistic tendencies (*Autism-Spectrum-Quotient*). In the *Symptom Check List (SCL-90R)* ML reported little tendency to somatize his problems, denied uncertainty in social situations or experiencing feelings of anxiety or inadequacy. In the *FPI-R*, ML answered that he had reduced life satisfaction, few physical complaints, very little aggressiveness, and perceived himself as being reserved and introverted. He experienced himself as being passive and with decreased motivation for achievement. On the openness subscale of *FPI-R*, ML scored significantly lower than his age group, seeming therefore to be very concerned with social conventions and social desirability. The subscale “openness” (willingness to admit minor weaknesses and violations of everyday conventions versus orientation toward making a socially desirable impression/social norms) of the *FPI-R* acts as a validity scale. Low results on this sub-scale point to socially desirable response tendencies. If a low result (stanines one to three) is reached in this subscale, interpretation of all other responses is limited (Fahrenberg et al., 2001; Fujiwara et al., 2008). According to findings from the self administration of *Toronto Alexithymia Scale (TAS-26*) ML did not experience any kind of difficulties with perceiving his own feelings and their accompanying bodily sensations. He scored low on the subscale for external orienting thinking style and perceived himself as being very interested in finding solutions in problematic situations. According to his self assessment, his ability to describe his own feelings was within normal limits. Given the deficient performance of ML on some tasks that objectively assessed emotional processing, one might argue that the results from self-evaluation scales or inventories should be interpreted cautiously. This is one of the reasons why we chose to ask both ML and his mother to complete the personal adjectives self-questionnaire (EWL-N). Both the mother and the patient confirmed that ML experienced no fear and no aggression. Out of 161 adjectives, 33 were judged differently by the two. ML's mother perceived ML as having no symptoms of depression and ML denied feeling depressed. While ML's mother perceived ML as being unconcentrated, undecided, and contemplative, ML viewed himself as being well concentrated, capable of decision making, and rejected all adjectives describing a contemplative or daydreaming nature. We speculate that ML's mother's perception of ML as being non-concentrated and a daydreamer might represent a misinterpretation of his memory difficulties (Gadian et al., 2000).

ML's capacity for empathy appeared even above average as assessed by one scale (*Cambridge Behavior Scale*). In the *Saar-brücken Personality Questionnaire* the only deviant scale score was that for personal distress. With a score of 85 on the latter scale ML supposedly experiences a lower (below average) level of personal distress (self oriented unpleasant feelings) when he is confronted with the distress of others. This scale's queries tap on aspects of emotional regulation. The low level of personal distress when confronted with the distress of others may also be corroborated with ML's view of himself as being interested in finding solutions in problematic situations. This pro-active attitude may be fueled by a decreased level of aversiveness or of conscious fear when confronted with various extreme situations, including the distress of others, which may be related to an amygdala dysfunction. Personal distress elicited by the distress of others is a self oriented motivated response directed toward self regulation of overarousal, which may be associated with fearfulness and emotional vulnerability (Cheetham et al., 2009). Too much personal distress might hinder the capacity for empathic concern; the amygdala and its connections seem to modulate the balance between the two (Feinstein et al., 2011; Roth-Hanania et al., 2011). The decreased level of aversiveness (probably due to an amygdalar dysfunction) may also account for the results on the approachability task, which were discussed above and for the behavior during administered variations of the economic games.

ML's scores on the fantasy scale, perspective-taking scale, and empathic concern scale fell within the average range (90–110), with the highest score (104) being achieved on the perspective-taking scale. Findings of average scores on the fantasy scale support its relationship with measures of verbal intelligence (Shamay-Tsoory et al., 2009). The perspective-taking scale was repeatedly related to interpersonal functioning, increased self esteem and social competence (Shamay-Tsoory et al., 2009).

The conduction of a psychiatric interview in conformity with DSM-IV-TR (2000) did not provide evidence of meeting full diagnostic criteria for a concomitant major depressive disorder, dysthymic disorder, or bipolar disorder (or other psychiatric disorder, with the exception of adjustment disorder). No substance use disorder was diagnosed. ML did not meet diagnostic criteria for a pervasive developmental disorder. [However, in line with the results of a new small study showing that some individuals with autism spectrum disorder may lose their diagnosis, one could argue that ML might have outgrown a range of symptoms later on (Fein et al., 2013).]

## Discussion

According to our knowledge, this is one of the first reports about a comprehensive evaluation of social information processing in a patient with amnesia with neurodevelopmental mechanisms (probable developmental amnesia). ML has a long standing history of difficulties with acquiring EAMs for long-term storage and with everyday memory performance (Vargha-Khadem et al., 1997, 2003; Willoughby et al., 2012). These impairments occurred in the context of postnatal hypoxia and reduced hippocampal formation volumes bilaterally. The reduction in hippocampal volumes likely happened due to the hypoxic incident; as mentioned above, a bilateral enlargement of the lateral ventricles (which suggests hippocampal volume reductions; Calabrese and Penner, 2007) had been noted more than 10 years prior to ML's most recent MRI imaging. Our voxel-based morphometry data confirmed markedly reduced gray-matter density within the hippocampus bilaterally, with an anterior and right-sided preponderance. Only when we used a whole brain analysis with a less conservative statistical threshold (*p* < 0.001, uncorrected), we identified further regions showing reductions of gray matter, such as both amygdalae and bilateral striatum and pallidum, bilateral thalamus (pulvinar) – again with a right-sided preponderance. Our findings partly overlap with the voxel-based morphometry results of Vargha-Khadem et al. (2003). One potentially intriguing result in our study is the reduction in the amygdala gray matter, when using a less conservative statistical threshold. The lack of reports of amygdala gray matter reductions in previous studies might be accounted for by the statistical threshold applied and/or the fact that apart from hippocampal formation, other medio-temporal lobe regions did not undergo rigorous volumetric quantifications (Vargha-Khadem et al., 2003; Rosenbaum et al., 2011). In line with other studies showing damage to thalamic areas after episodes of (perinatal) hypoxia (Jacob and Pyrkosch, 1951; Voit et al., 1987; Markowitsch et al., 1997; Cowan et al., 2003; Macey et al., 2005; de Haan et al., 2006), we identified indices of reductions in gray matter density in thalamus, namely in the pulvinar, when we applied a less conservative statistical threshold (*p* < 0.001, uncorrected).

The right-sided preponderance of the identified structural brain changes is relevant, given data suggesting a right hemispheric bias for emotional processing (Schore, 2002), EAM (Fink et al., 1996; Markowitsch et al., 2000) and “high-order consciousness” (Keenan et al., 2005) (however, for different results, see Nyberg et al., 2010; Viard et al., 2012).

On the behavioral level, ML shared several similarities with patients with developmental amnesia, but also displayed some distinct features (such as atypical school trajectory, different level of functional independence). He showed evidence of at least average intelligence; in some measures of intelligence his performance even was substantially above the normal level. His attention and concentration abilities were at least average and for some tests, above average. The same held true for executive functions and problem solving abilities, which were to a large degree within normal range. In the *Concept Comprehension Test* (Cronin-Golomb et al., 1987a,b) ML showed intact performance for concrete concepts, but sub-average for abstract concepts (Martins et al., 2006; Quian Quiroga, 2012). This result is congruent with data suggesting a role of hippocampal formation in facilitation of more complex problem solving, via comparison computations (Olsen et al., 2012). In the TkS, ML showed impairments comparable to those of patients with Korsakoff's syndrome (Brand et al., 2003a). These results may have to do with the test's specification. As mentioned above, deficits in the TkS time items had been conjectured to depend on timing deficits combined with remote memory impairment (Brand et al., 2003a).

ML's major and in fact very severe problems were identified in the domains of long-term memory. This was evident in anterograde memory tests where he almost did not recall anything after half an hour delay. It was – if this comparison is possible – even more evident when it came to EAM recall. ML was completely unable to provide authentic memories for personal events, aside from one, where his father jumped out of the window in a suicide attempt. The recall of this extraordinary event can be compared with the islands of knowledge that had been found in patient H.M. of Scoville and Milner (1957) and Corkin (2002). (After surgical removal of his medial temporal lobe regions bilaterally, H.M. was completely anterogradely amnesic. Exceptions were that he could recall the death of his parents, the killing of President Kennedy, and a particular song. All these were, however, semanticized memories; cf. Markowitsch, 1985.)

ML showed impairments in both semantic and episodic–autobiographical anterograde memory. He for example had no recollection of a previous meeting with one of his examiners (AS), although he admitted to a feeling of familiarity. He was able to recall factual aspects of his meetings with examiner SB, but these memories did not have an episodic quality. In particular they lacked contextual details and the subjective feeling of reliving. In contrast to his performance on various anterograde explicit memory tests, his old semantic knowledge was good, which suggests a use of compensatory strategies (Rosenbaum et al., 2011). His performance on free recall was worse than that on recognition which is consistent with findings from patients with developmental amnesia (Adlam et al., 2009). Adlam et al. (2009) reported a dissociation between recall and recognition; this was however incomplete, in the sense that their patients with developmental amnesia displayed (similarly to ML) a subtle, but significant visual recognition impairment in the Doors test. ML's poor performance on prospective memory tests mirrors data suggesting a role for hippocampal formation in prospection (however, see Markowitsch and Staniloiu, 2012 for controversial results). Prospective memory impairments have been linked not only to deficits in executive functions, but also to deficits in semantic memory and episodic-autobiographical memory (Hainselin et al., 2011). Apart from the hippocampal formation, the amygdala was also suggested to be involved in the simulation of future emotional events. In particular, a strong connectivity between rostral anterior cingulate cortex (ACC) and amygdala was observed during the imagination of positive future events (Sharot et al., 2007) and was postulated to underlie traits like optimism bias.

ML's performance on tasks that tap on social information processing abilities revealed both domains of preserved performance and impairment. In the *Florida (Tübingen) Affect Battery* ML performed well on simple tasks, but he displayed below average performance in the more complex subtests. Difficulties with emotional processing were also apparent on tests that assessed recognition and free recall of emotional stimuli. In the picture story for affect induction (BAd), his performance showed only a marginal benefit from the enhancing effect of emotional connotation. From a neurobiological perspective, ML's difficulties with emotional processing may be related to his gray matter reductions in hippocampal formation (with an anterior preponderance; Fanselow and Dong, 2010; Viard et al., 2012), and/or possible amygdala, basal ganglia (caudate), and pulvinar dysfunctions (Adolphs, 2010b; Markowitsch and Staniloiu, 2011b; Geschwind et al., 2012; Kemp et al., 2012; Nguyen et al., 2013; Saalmann and Kastner, 2013). Performance on affect-related memory tasks had been found to be impaired in patients with bilateral amygdala calcifications due to a genetic disorder (Urbach-Wiethe disease), compared to normal participants (Sarter and Markowitsch, 1985a,b; Cahill et al., 1995; Siebert et al., 2003). Similarly to the amygdala (Markowitsch and Staniloiu, 2011b), the pulvinar has been postulated to play a role in detecting salience (van Buren and Borke, 1972; Pessoa, 2011) and, due to its extensive connections with cortical areas, in the processing of emotional material (Pessoa, 2008, 2011; Pessoa and Adolphs, 2010). The hippocampal formation has been attributed a role not only in cognition, but also in emotional processing. In particular, the anterior hippocampus (which was significantly affected in our patient) has been assigned a function in emotional processing, reward, goal proximity, and arousal (Fanselow and Dong, 2010; Viard et al., 2012); the nature of functional specialization within the hippocampal formation in particular (and medio-temporal lobe, in general) is yet not a solved issue, however (Chua et al., 2007; Markowitsch and Staniloiu, 2012). Furthermore, it has been proposed that the function of the hippocampus extends beyond the mnemonic domain, to support other cognitive areas (such as perception, problem solving) via relational binding and comparison (Abu-Akel and Shamay-Tsoory, 2011; Olsen et al., 2012).

Some authors hypothesized that the hippocampal formation may play a role in the experience of emotions about others' mental states (Immordino-Yang and Singh, 2013). Negative correlations were found in a study between vocally expressed nervousness and regional cerebral blood flow in the right hippocampus (Laukka et al., 2011). Right temporal cortices (including right anterior temporal structures), the right thalamus, and also the amygdala and orbitofrontal cortices have been involved in affective-prosodic comprehension by some studies (Ross and Monnot, 2011). Additionally, basal ganglia have at times been implicated in emotional prosody decoding (Paulmann et al., 2008; Frühholz et al., 2012). While some authors had linked affective prosody decoding with the right hemisphere, several others have challenged this view (for a review, see Snitz et al., 2002; Frühholz et al., 2012). It is perhaps worth mentioning that the performance of ML on affective prosody tasks resembled in several respects that of the seven patients with Korsakoff syndrome, who were examined by Snitz et al. (2002). Those patients showed in comparison to control participants impairments in naming affective prosody when semantic content was incongruent, while both linguistic and affective prosody discriminations were intact. The authors did not provide a neurobiological explanation for their findings, though they made speculations about the involvement of the basolateral circuit.

On several standard laboratory tests for ToM, ML showed normal performance or only mild impairment. These results are consistent with largely preserved core ToM functions: however, our findings are slightly different from those of Rabin et al. (2012) who reported normal performance on standardized tests for ToM in an adult woman with developmental amnesia. There are several factors that may account for the observed differences, such as the use of different test adaptations (e.g., the German adaptation of the RMET) and the presence of neural correlates' and sex differences (Schulte-Rüther et al., 2008; Frank, 2012; Kemp et al., 2012; Nguyen et al., 2013; Saalmann and Kastner, 2013). In particular, the imaging study of Frank (2012) found that young men recruit during a false belief task more brain regions known to be implicated in episodic memory than young women.

In the Heider and Simmel animation paradigm ML partly deviated in his responses from those of comparison groups. Contrary to controls he did not use animated nouns and gave no description of internal states (Ross and Olson, 2010). On the other hand, self questionnaires yielded no indications of difficulties with empathy or perspective-taking apart from the Saarbrücken Personality Questionnaire (personal distress scale). As commented above, low personal distress elicited by the distress of others may reflect decreased conscious fearfulness (Cheetham et al., 2009), perhaps in the context of an anterior hippocampus and/or amyg-dalar dysfunction (Maren and Holt, 2004; Fanselow and Dong, 2010; Feinstein et al., 2011). Incidentally, on the EWL-N [List of personal adjectives] (Janke and Debus, 1978), both ML's mother and ML consistently rejected adjectives synonyms to “fearful” as applying to ML.

ML's results on the approachability task could be interpreted as evidence of increased sociability and/or decreased aversiveness. He tended to overrate (both negative and positive) faces as being approachable. These findings corresponded to observations from ML's everyday life behavior. From a neurobiological perspective, an interaction between insula (which has bidirectional connections to amygdala) and hippocampus was found to be important for processing of untrustworthy faces (Tsukiura et al., 2012). Furthermore, decreased aversiveness and/or hypersociability have been related to various amygdala dysfunctions (Bellugi et al., 1999; Martens et al., 2009). It is possible that the hypersociability may stem in ML's case from decreased fearfulness/aversion, which may be underlain by an anterior hippocampus and/or amygdalar dysfunction (Maren and Holt, 2004; Fanselow and Dong, 2010; Feinstein et al., 2011).

No tendency for risky behavior was identified in ML by his performance in the Game of Dice Task. This finding is in some contradiction with his increased trustworthiness leading him to engage in potentionally risky or dangerous behaviors.

Tests of malingering partly yielded results which – for an individual with normal conscious mnemonic processing abilities – would be indicative of feigning. As ML was quite deficient in memory recall of new material in general, these test outcomes cannot be interpreted as indicative of feigning. Based on our objective multidimensional assessment and the collateral information we obtained, we concluded that it was extremely unlikely that ML was trying to feign his impairments (either for an external incentive, such as in malingering or for deliberately trying to assume the sick role).

## Conclusion

Episodic–autobiographical memory contains a wealth of information about people and social interactions; this led several authors to hypothesize that the exchange of EAM might facilitate social cognition, such as understanding of others' inner world and perspective (Nelson and Fivush, 2004; Spreng et al., 2008) and subsequently might connect and “draw the world together” (Casey, 2000, p. 313). In a review article, Abu-Akel and Shamay-Tsoory (2011) argued that, while primarily perception-based, the representation of mental states of others might also be computed by resorting to internally stored information such as from episodic-autobiographical memories. Data coming from a study of healthy participants suggested that laboratory ToM tasks engage more cognitively economical ways than accessing own EAMs (Rabin et al., 2010), relying on semantic knowledge and/or implicit processing (Adolphs, 2010a; Staniloiu et al., 2010b). Yet another study, which investigated the role of hippocampus in emotional mentalizing, proposed that mentalizing is modulated by memories of past events (Perry et al., 2011). Interesting data came from a recent study of Rabin and Rosenbaum (2012), which showed that in healthy female participants the functional relation between autobiographical memory and ToM is modulated by the familiarity of the target person in a ToM task, which in turn affected the employment of cognitive strategies.

Herein we used a broad variety of tests and found objective evidence of intact areas as well as impaired domains of social information processing in a young adult male patient with amnesia with neurodevelopmental mechanisms, which preponderantly afflicted episodic–autobiographical mnemonic processing. Consistent with data from other authors (Rabin et al., 2012), we identified in ML's case largely preserved ability to perform on a number of tasks testing core ToM functions; this is in line with suggestions that neither intact EAM nor an intact hippocampal formation are essential for core social cognitive processes (Rosenbaum et al., 2007; Rabin et al., 2012). Impairments were however detected in ML's case on certain tasks taping on complex social perception or complex social judgments. These results could be interpreted in different ways. The hippocampal formation has been attributed roles in relational binding (flexible association of disparate items) and comparison computations (Olsen et al., 2012). The hippocampus-related relational binding was proposed to underlie constructive processes linked to EAM, mental scene construction, and self-projection (Spreng et al., 2008; Addis et al., 2009; Rosenbaum et al., 2009; Spreng and Grady, 2010) (but, see McKinlay et al., 2009; Squire et al., 2010 for different opinions). Furthermore, hippocampus-mediated constructive processes were suggested in a recent study to be implicated in both EAM and ToM tasks targeting familiar individuals (Rabin and Rosenbaum, 2012). An interesting idea was however put forth by Olsen et al. (2012). The authors stated: “Thus, while binding and comparison computations may be mediated by multiple neural regions, the hippocampus is critical for these computations when relational information must be maintained over longer delays and/or when information has a high degree of conceptual/perceptual overlap, regardless of whether the information must be maintained over a delay or merely discriminated in the present moment” (Olsen et al., 2012, p. 10). Along this vein we could argue that our findings support ideas that an intact hippocampal formation might be necessary for adequate performance on tasks that require demanding, complex (perceptual) information processing, involving complex relational information and/or a need for longer online holding of complex relational information (Olsen et al., 2012).

Alternative explanations for our results may be linked to our voxel-based morphometry data, which pointed to indices of reduced gray matter in amygdala, basal ganglia, and pulvinar, when a less conservative threshold was used. All these structures were described to make contribution to social cognition and social information processing (Jacobson, 1986; Adolphs et al., 1998, 1999, 2005; Shaw et al., 2004; Pessoa, 2008, 2011; Adolphs, 2010a,b; Bodden et al., 2010a,b; Pessoa and Adolphs, 2010; Kemp et al., 2012; Nguyen et al., 2013; Saalmann and Kastner, 2013). Furthermore, several of these structures were reported to be affected in various memory disorders (Zingerle, 1912; Schuster, 1936; Smyth and Stern, 1938; Stern, 1939; Guard et al., 1986; Kuljis, 1994; Mori et al., 1999; Siebert et al., 2003; Allen et al., 2006). While white matter changes were not detected on visual inspection in the case of ML, subtle white matter changes due to hypoxia cannot be ruled out and would require more refined imaging techniques in order to be captured (Paus, 2010). Their detection may however be relevant for a number of reasons: there are numerous reports on amnesia arising after white matter lesions (Horel, 1978; Zola-Morgan et al., 1982; Markowitsch et al., 1990; Calabrese et al., 1995; Markowitsch and Staniloiu, 2012); furthermore, there are some reports of ToM impairments occurring after white matter tract damage (Bach et al., 1998; Happé et al., 2001).

Due to their non-uniformity, our findings draw attention to the need of employing several tests for assessing social information processing. Furthermore, they call for testing social cognition in real life settings or conditions that approximate real life settings, in order to fully assess the extent of disability and appreciate any contribution that EAM may play to understanding the inner world of others and “drawing the world together” (Casey, 2000, p. 313).

### Conflict of interest statement

The authors declare that the research was conducted in the absence of any commercial or financial relationships that could be construed as a potential conflict of interest.
